# Hybrid surgical thromboembolectomy and crossover endovascular revascularization for acute lower-limb ischemia in a patient with active ovarian cancer receiving a PARP inhibitor (fuzuloparib): a case report and literature review

**DOI:** 10.3389/fcvm.2026.1916588

**Published:** 2026-10-07

**Authors:** Yibiao Jiang, Gang Wang, Zhenbin Liu, Haoran Jiao, Bowen Wang

**Affiliations:** 1 Department of Wound and Peripheral Vascular Surgery, First Teaching Hospital of Tianjin University of Traditional Chinese Medicine, Tianjin, China; 2 National Clinical Research Center for Chinese Medicine, Tianjin, China

**Keywords:** acute limb ischemia, anticoagulation, atherosclerosis, cancer-associated arterial thrombosis, fuzuloparib, hybrid revascularization, ovarian cancer, PARP inhibitor

## Abstract

Acute limb ischemia (ALI) is both limb- and life-threatening, and represents a vascular emergency. Malignancy produces a systemic hypercoagulable state that predisposes to arterial as well as venous thrombosis, and poly(ADP-ribose) polymerase (PARP) inhibitors—now central to maintenance therapy in ovarian cancer—have, in aggregate randomized data, been associated with only a borderline venous signal and no clearly demonstrated independent arterial signal. We report a 60-year-old woman with active high-grade serous ovarian carcinoma maintained on oral fuzuloparib who presented with Rutherford category IIa ALI of the right lower limb on a background of chronic bilateral atherosclerosis. Angiography demonstrated occlusion of the right common iliac–external iliac–common femoral–proximal superficial femoral segment with collateral reconstitution and, after dedicated cardiac evaluation (regular sinus rhythm on ECG, no arrhythmia on perioperative telemetry, normal transthoracic echocardiography without intracardiac thrombus or vegetation, and no critical carotid stenosis), no identifiable cardioembolic source. A single-stage hybrid procedure—open common femoral thromboembolectomy followed by contralateral (crossover) endovascular treatment of a residual flow-limiting dissection with overlapping self-expanding and balloon-expandable covered stents—restored inline flow and salvaged the limb; the retrieved material was submitted for histopathology, which demonstrated bland fibrin–platelet–red-cell thrombus with lines of Zahn and negative AE1/AE3 immunostaining, excluding tumour embolism. The patient was transitioned to therapeutic anticoagulation within a multidisciplinary framework, and at 3 months the limb remained viable with palpable distal pulses. This case illustrates cancer-associated arterial thrombosis superimposed on atherosclerosis—on a background that also included PARP-inhibitor exposure, whose independent contribution remains speculative—highlights a hybrid open–endovascular strategy for definitive single-setting revascularization, and underscores the importance of excluding a cardioembolic source and confirming thrombus nature histologically, while individualizing long-term anticoagulation that is necessarily extrapolated from cancer-associated venous thromboembolism evidence, with attention to drug–drug interactions, thrombocytopenia and bleeding risk.

## Graphical Abstract

**Figure:**
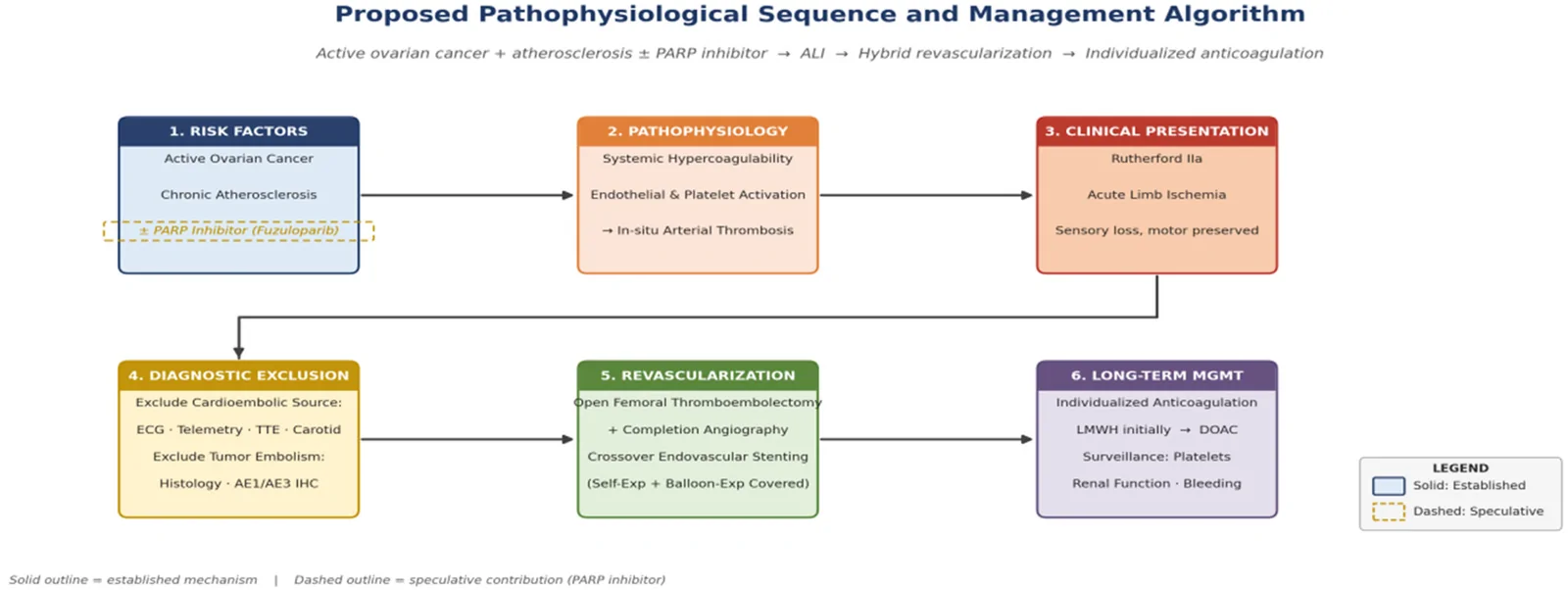
Proposed pathophysiological sequence and management algorithm for the present case. Active ovarian cancer with chronic atherosclerosis (with a speculative, non-causal contribution from PARP-inhibitor exposure) → systemic hypercoagulability plus local endothelial/platelet activation → *in-situ* arterial thrombosis of the right iliofemoral–superficial femoral segment → Rutherford IIa acute limb ischemia (sensory loss with preserved motor function) → systematic exclusion of a cardioembolic source (ECG, telemetry, transthoracic echocardiography, carotid duplex) and histological exclusion of tumor embolism (negative AE1/AE3 immunostaining on retrieved thrombus) → single-setting hybrid revascularization (open common femoral thromboembolectomy → completion angiography → contralateral crossover endovascular treatment of flow-limiting dissection with overlapping self-expanding and balloon-expandable covered stents) → individualized long-term antithrombotic therapy (LMWH initially, with multidisciplinary consideration of DOAC transition) under continued surveillance of perfusion, platelet count, renal function, drug–drug interactions, and bleeding risk. Elements rendered in dashed outline indicate the contribution of PARP-inhibitor exposure, which remains speculative and cannot be causally inferred from a single case. Solid-outline elements represent the components that, in this patient, were sufficient to explain the event and that should be the principal targets of clinical attention and future prospective study.

## Introduction

Acute limb ischemia (ALI) is defined as a sudden decrease in limb perfusion that threatens limb viability, generally of less than two weeks’ duration. Population data suggest an incidence of roughly 1–2 per 10,000 person-years, and ALI remains both limb- and life-threatening: despite urgent revascularization, in-hospital mortality and one-year mortality remain substantial, particularly in frail or comorbid patients (1). The two dominant mechanisms are embolism—classically cardiac, for example from atrial fibrillation—and *in-situ* thrombosis, which may occur on an atherosclerotic plaque, within a bypass graft or stent, or in the setting of a systemic hypercoagulable state (1, 3).

Malignancy is among the most important acquired causes of hypercoagulability. The association between cancer and thrombosis, first articulated by Trousseau, is now understood to arise from tumor-cell expression of procoagulants, activation of platelets and the coagulation cascade, and impaired fibrinolysis (5–7). Although venous thromboembolism (VTE) dominates the clinical picture, arterial thromboembolism (ATE)—including myocardial infarction, ischemic stroke and peripheral arterial events—is also significantly increased in patients with cancer, particularly in the months surrounding diagnosis, and carries an approximately three-fold excess mortality (9, 10). Among solid tumors, the mucin-producing adenocarcinomas of the ovary, pancreas and gastrointestinal tract are especially thrombogenic (6, 7).

Poly(ADP-ribose) polymerase (PARP) inhibitors have transformed the maintenance treatment of high-grade ovarian carcinoma, conferring substantial improvements in progression-free survival, particularly in tumors with BRCA mutation or homologous-recombination deficiency. Fuzuloparib (formerly fluzoparib) is the first domestically developed oral PARP inhibitor approved in China, indicated for platinum-sensitive recurrent ovarian cancer and as maintenance therapy in newly diagnosed advanced and platinum-sensitive recurrent disease (13, 14). A growing body of evidence, including a 2025 meta-analysis of 38 randomized trials (15,008 patients), indicates that PARP inhibitors carry a thrombotic signal whose magnitude is modulated by tumor subtype and concurrent therapy; notably, the overall venous signal was borderline and was driven mainly by prostate and pancreatic cancer, with no statistically significant increase in the ovarian subgroup (11, 12).

The convergence of active malignancy and PARP-inhibitor exposure—here superimposed on pre-existing atherosclerotic disease—nevertheless represents a particularly prothrombotic state, yet ALI in this specific context is rarely described, and there is little guidance on the choice of revascularization technique or the duration and agent of long-term anticoagulation. We report a patient with active ovarian cancer on fuzuloparib who developed ALI, was successfully treated with a hybrid open thromboembolectomy and crossover endovascular revascularization, and remained well at 3 months, and we review the relevant literature with emphasis on cancer-associated arterial thrombosis, PARP-inhibitor–related thrombotic risk, and the resulting long-term anticoagulation considerations. The report is presented in accordance with the CARE guidelines.

## Case presentation

### History and oncologic background

A 60-year-old woman presented to the emergency department with the sudden onset, 9 h earlier, of swelling, coldness and numbness of the right lower limb, which progressively worsened. Her relevant history was active high-grade serous ovarian carcinoma diagnosed approximately two years previously, treated with total hysterectomy and bilateral salpingo-oophorectomy (2024, at another institution) and platinum-based chemotherapy, and currently maintained on oral fuzuloparib 150 mg twice daily (FIGO stage IV). She also had a 10-year history of an anxiety disorder, currently managed on a multi-drug home regimen consisting of olanzapine 5 mg orally at bedtime, trazodone 50 mg orally at bedtime, clonazepam 0.5 mg orally twice daily, and venlafaxine 75 mg orally once daily. Pre-operative pharmacokinetic screening for drug–drug interactions involving cytochrome P450 3A4 (CYP3A4) and P-glycoprotein (P-gp) was performed when selecting long-term anticoagulation. Each of the four psychotropics is itself a substrate of CYP3A4 and/or P-gp—olanzapine is metabolized principally by CYP1A2 with contributory CYP3A4- and CYP2D6-mediated clearance and is a P-gp substrate; trazodone is N-dealkylated by CYP3A4 to its active metabolite m-chlorophenylpiperazine and is both a CYP3A4 substrate and a weak P-gp substrate; venlafaxine undergoes O-demethylation by CYP2D6 to desvenlafaxine with secondary CYP3A4-mediated N-demethylation and is a recognized P-gp substrate; and clonazepam is extensively nitroreduced by CYP3A4 and is a P-gp substrate. None is a clinically relevant strong inducer or inhibitor of CYP3A4 or P-gp, although trazodone may exert modest weak CYP3A4 inhibition at higher doses. Candidate long-term anticoagulants—DOACs (rivaroxaban and apixaban, dual CYP3A4/P-gp substrates; edoxaban and dabigatran, P-gp-only substrates), low-molecular-weight heparin (LMWH, e.g., enoxaparin, eliminated via renal and reticuloendothelial routes independent of CYP450 and P-gp), and warfarin (a racemic mixture metabolized predominantly by CYP2C9 with secondary contributions from CYP3A4, CYP1A2, and CYP2C19)—were systematically screened. Because the four psychotropics are themselves CYP3A4/P-gp substrates rather than potent inhibitors or inducers, the principal risk is not a clinically meaningful change in their own plasma concentrations by the anticoagulant, but rather the converse: any concurrent potent CYP3A4 and/or P-gp inhibitor (e.g., ketoconazole, itraconazole, ritonavir, clarithromycin) or inducer (e.g., rifampin, carbamazepine, phenytoin, St John's wort) could require dose adjustment of rivaroxaban or apixaban, and any potent P-gp inhibitor or inducer could alter the exposure of edoxaban or dabigatran. In this patient's stable outpatient regimen, however, no concomitant strong CYP3A4 or P-gp perpetrator was identified, and the four anxiolytic/antidepressant agents are not expected to alter the pharmacokinetics of rivaroxaban, apixaban, edoxaban, dabigatran, warfarin, or LMWH to a clinically significant degree. Accordingly, from a strict drug–drug interaction perspective, the choice of long-term anticoagulant was not constrained by CYP3A4- or P-gp-mediated interactions with the home psychiatric regimen; the final selection was instead driven by oncologic guidelines, bleeding risk, renal function, drug–drug interactions with fuzuloparib, gastrointestinal/genitourinary bleeding risk from the pelvic ovarian disease, and the need for parenteral therapy in the immediate peri-revascularization period. She denied hypertension, coronary artery disease, diabetes mellitus, hyperlipidemia, and prior stroke, and had no known atrial fibrillation; however, duplex ultrasonography demonstrated bilateral lower-limb arteriosclerosis, indicating underlying chronic peripheral arterial disease. She was ambulatory at baseline.

### Examination and rutherford classification

On examination the right lower limb was mildly swollen, pale and cool with reduced sensation; active movement was preserved. Chronic trophic changes were present—rough, scaly skin, sparse and lustreless hair, and brittle, thickened, ridged toenails—consistent with antecedent chronic ischemia. On the right, the femoral, popliteal, dorsalis pedis and posterior tibial pulses were all absent; on the left, the corresponding pulses were diminished (weak) rather than normal, again reflecting bilateral arterial disease. These findings were consistent with Rutherford category IIa acute limb ischemia (sensory loss with preserved motor function) (1, 2). The ABI of the affected limb was unobtainable; the contralateral ABI was 0.98.

### Laboratory, cardiac rhythm and echocardiographic evaluation

Laboratory testing showed a markedly elevated D-dimer of 8.6 mg/L FEU and fibrinogen of 5.8 g/L, with normal prothrombin time and activated partial thromboplastin time and a platelet count of 178 × 10⁹/L. CA125 was 320 U/mL, consistent with active disease.

Because a cardioembolic mechanism would substantially alter both attribution and management, dedicated cardiac evaluation was undertaken. A 12-lead electrocardiogram on admission showed regular sinus rhythm at 76 bpm without atrial fibrillation, atrial flutter, ventricular arrhythmia, conduction abnormality or evidence of acute myocardial ischemia. Continuous inpatient telemetry throughout the perioperative period revealed no atrial fibrillation, atrial flutter, sustained supraventricular or ventricular arrhythmia, or new conduction block. Transthoracic echocardiography demonstrated preserved biventricular systolic function (left ventricular ejection fraction 62%), normal left ventricular wall motion without regional wall-motion abnormality suggestive of prior infarction, normal cardiac chamber dimensions, and—importantly—no left atrial or left ventricular thrombus, no valvular vegetation, and no intracardiac mass lesion; mild mitral and tricuspid regurgitation without hemodynamic significance was noted, and the aortic valve was tricuspid without significant stenosis or regurgitation. Carotid duplex ultrasonography demonstrated non-hemodynamically significant bilateral atheromatous plaques without critical stenosis. Collectively, no clinical, electrocardiographic, rhythm-monitoring, echocardiographic, or carotid-imaging evidence of a cardioembolic source was identified, strengthening the inference of *in-situ* cancer-associated arterial thrombosis superimposed on chronic atherosclerosis. Detailed laboratory and clinical parameters across the treatment course are summarized in Table 1.

**Table 1:** Longitudinal clinical and laboratory parameters across the treatment course.

Parameter (unit)	Reference range	Admission/pre-op	POD 1	1 month	3 months
ABI, affected limb	>0.90	(unobtainable)	0.55	0.78	0.92
ABI, contralateral limb	>0.90	0.98	0.97	0.98	0.99
Rutherford ALI category	—	IIa	Resolved (I)	—	—
D-dimer (mg/L FEU)	<0.50	8.6	4.2	1.1	0.4
Fibrinogen (g/L)	2.0–4.0	5.8	4.9	3.6	3.1
PT (s)	11.0–14.5	12.8	13.0	13.1	13.0
APTT (s)	25–37	28	62 (on UFH)	30	29
INR	0.8–1.2	1.04	1.05	1.06	1.05
Anti-Xa, peak (IU/mL)	0.6–1.0 (Rx)	—	0.92	0.98	0.95
Platelet count (×10⁹/L)	125–350	178	146	132	158
Hemoglobin (g/L)	115–150	112	98	106	119
CA125 (U/mL)	<35	320	286	150	102
Creatinine clearance (mL/min)	>90	82	78	85	88

ABI, ankle–brachial index; APTT, activated partial thromboplastin time; FEU, fibrinogen-equivalent units; INR, international normalized ratio; POD, postoperative day; PT, prothrombin time; Rx, therapeutic target; UFH, unfractionated heparin. The Rutherford category applies to the ischaemic limb at presentation and is not used after revascularization.

**Table 2:** Summary of selected, source-verified evidence on acute limb ischemia, cancer-associated arterial thrombosis, PARP-inhibitor thrombotic risk, and anticoagulation guidance.

Source (first author, year, journal)	Design	Population (N)	Key thrombosis-related finding
A. Acute limb ischemia: management and outcomes (including cancer)			
Björck M et al., 2020 (ESVS CPG; Eur J Vasc Endovasc Surg) (1)	Clinical practice guideline	Acute limb ischaemia (all aetiologies)	Rutherford grade directs therapy; open thromboembolectomy, catheter-directed thrombolysis, percutaneous mechanical thrombectomy and hybrid (open+endovascular) approaches are all options; completion angiography is a key adjunct; revascularisation is reasonable in selected patients with malignancy (Class IIa/B); hypercoagulable states raise the risk of distal microvascular thrombosis and minor amputation after thrombectomy.
Govsyeyev N et al., 2021 (J Vasc Surg) (4)	Systematic review/meta-analysis (7 studies, 2,899 patients)	Patients with cancer and ALI	Genitourinary, lung, gastrointestinal and skin/soft-tissue cancers accounted for most ALI events; 1-year mortality after ALI exceeded 50%; an underlying cancer should be considered when ALI aetiology is unclear.
Taha AG et al., 2024 (J Endovasc Ther) (29)	Prospective single-centre series	Native-vessel acute lower-limb-threatening ischaemia (Rutherford II)	Image-guided surgical thromboembolectomy with routine intraoperative completion angiography and selective endovascular adjuncts achieved high technical success and limb salvage—supporting routine completion imaging and a hybrid strategy.
B. Cancer-associated arterial thrombosis: epidemiology and mechanism			
Navi BB et al., 2017 (J Am Coll Cardiol) (10)	Matched cohort (SEER–Medicare)	Solid-tumor cancer vs. matched controls	6-month cumulative arterial thromboembolism markedly elevated with cancer (e.g., myocardial infarction 2.0% vs. 0.7%; ischaemic stroke 3.0% vs. 1.6%); risk highest in the period around diagnosis.
Grilz E et al., 2018 (Haematologica) (9)	Prospective cohort	Patients with cancer	Arterial thromboembolism independently associated with an approximately 3-fold increase in mortality.
Falanga A 2023 (J Thromb Haemost) (6); Hisada Y & Mackman N 2017 (Blood) (7)	Mechanistic/biomarker reviews	Cancer-associated thrombosis	Tissue factor and tissue-factor–bearing extracellular vesicles, podoplanin, neutrophil extracellular traps and mucin–selectin interactions drive both venous and arterial thrombosis; D-dimer and related biomarkers track thrombotic burden.
C. PARP-inhibitor thrombotic risk (and fuzuloparib efficacy context)			
Yazgan SC et al., 2025 (ESMO Open) (11)	Meta-analysis (38 RCTs, 15,008 patients)	Solid tumors on PARP inhibitors	Borderline overall VTE signal (any-grade VTE 2.4% vs. 1.6%; OR 1.37, 95% CI 1.00–1.88, *P* = 0.050) driven mainly by prostate and pancreatic cancer; no significant increase in the ovarian subgroup (11 trials, *n* = 4,348).
Sánchez Cánovas M et al., 2026 (SEOM; Clin Transl Oncol) (12)	Multicentre retrospective (real-world)	Ovarian cancer on PARP inhibitors (329 patients, 2015–2022)	VTE/ATE incidence 4.9% (16/329); BRCA2 mutation more prevalent among those with events (56.3% vs. 19.2%, *p* < 0.001); concomitant bevacizumab associated with lower thrombosis (OR 0.262, 95% CI 0.095–0.724, *p* = 0.010).
Wei Q et al., 2022 (Front Oncol) (15)	Case report	BRCA-mutant advanced HGSOC on niraparib	First reported pulmonary embolism during niraparib maintenance, managed with anticoagulation—illustrates individual-patient thrombotic risk on PARP-inhibitor therapy.
Wu L et al., 2026 FZOCUS-1 (CA Cancer J Clin) (14); Li N et al., 2022 (J Gynecol Oncol) (13)	Phase 3 RCT/drug review	Newly diagnosed advanced ovarian cancer; fuzuloparib	Fuzuloparib monotherapy (150 mg BID) prolonged PFS vs. placebo (median 29.9 vs. 11.1 months; HR 0.58); adding apatinib gave no additional PFS benefit—establishes efficacy and the dosing context of the index drug.
D. Anticoagulation for cancer-associated thrombosis			
Farge D et al., 2022 ITAC (Lancet Oncol) (16)	International guideline	Cancer-associated thrombosis	LMWH or a DOAC for ≥6 months; avoid DOACs when gastrointestinal/genitourinary bleeding risk is high or strong drug–drug interactions exist.
Key NS et al., 2023 ASCO (J Clin Oncol) (17); Lyman GH et al., 2021 ASH (Blood Adv) (18)	Guidelines	Cancer-associated VTE	Initial LMWH or DOAC; suggest extended/indefinite anticoagulation while cancer is active or under systemic therapy, with periodic reassessment of benefit and bleeding risk.
Hokusai-VTE Cancer 2018 (20); SELECT-D 2018 (21); Caravaggio 2020 (22); CLOT 2003 (19)	Phase 3 RCTs	Cancer-associated VTE	DOACs non-inferior to dalteparin for recurrent VTE; edoxaban and rivaroxaban associated with more GI/GU bleeding, apixaban without significant excess major bleeding; CLOT established LMWH superiority over warfarin.

ALI, acute limb ischemia; ATE, arterial thromboembolism; CPG, clinical practice guideline; DOAC, direct oral anticoagulant; GI, gastrointestinal; GU, genitourinary; HGSOC, high-grade serous ovarian carcinoma; LMWH, low-molecular-weight heparin; PFS, progression-free survival; RCT, randomized controlled trial; VTE, venous thromboembolism. Reference numbers in brackets correspond to the reference list.

### Imaging

Emergency duplex ultrasonography demonstrated arteriosclerosis with sluggish flow and findings consistent with occlusion of the distal right external iliac artery and common femoral artery; the deep and superficial veins of both lower limbs were patent with competent valves. Intra-operative digital subtraction angiography (DSA), performed via retrograde left brachial access, demonstrated occlusion of the right common iliac (distal)–external iliac–common femoral–proximal deep femoral–proximal/mid superficial femoral segment, with the mid–distal deep femoral artery reconstituted through fine internal-iliac collaterals and markedly sluggish flow in the distal superficial femoral, popliteal and tibial vessels (Figure 1). The angiographic appearance—an abrupt occlusion with surrounding fine collaterals—together with the absence of an identified cardioembolic source and the oncologic context, was most consistent with cancer-associated *in-situ* arterial thrombosis superimposed on chronic atherosclerotic disease rather than cardiac embolism (1, 3, 8).

**Figure 1:**
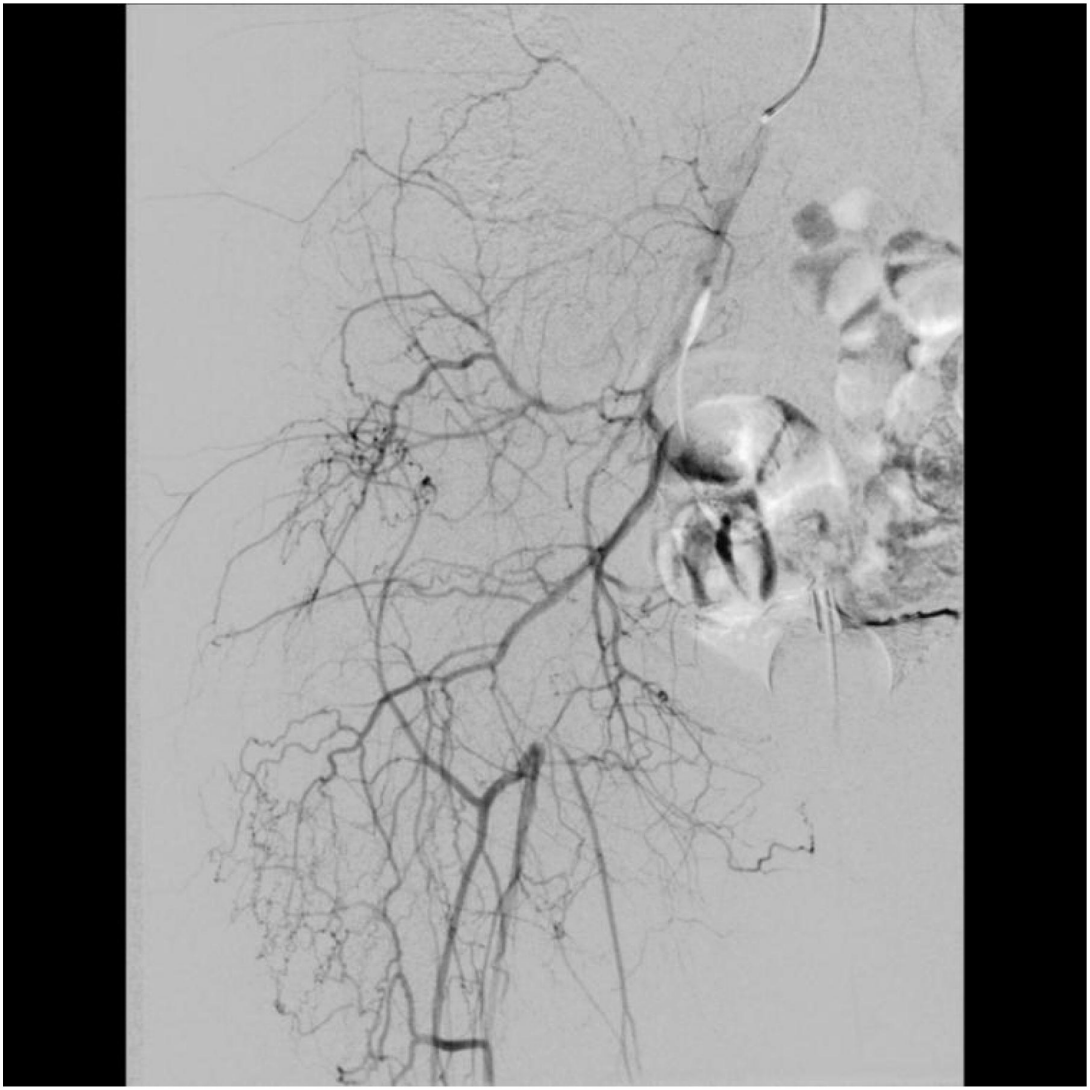
Pre-procedural/diagnostic digital subtraction angiography. Occlusion of the right common iliac (distal)–external iliac–common femoral–proximal superficial femoral segment with extensive collateral reconstitution and sluggish runoff.

### Treatment: hybrid open thromboembolectomy and crossover endovascular revascularization

Intravenous unfractionated heparin (UFH) was administered immediately (bolus followed by an infusion titrated to an activated partial thromboplastin time of 2–2.5 times control). Given the Rutherford IIa category, the need for rapid and definitive reperfusion, and the ability to treat residual thrombus and any underlying inflow/outflow lesion in a single setting, a hybrid strategy was chosen (1).

Diagnostic angiography was first performed via retrograde left brachial access (5-F sheath). Aortography (5-F pigtail catheter) and selective right common iliac angiography (5-F MPA catheter) confirmed the occlusion described above (Figure 1). Under local anaesthesia, the right common femoral artery was exposed through an approximately 8-cm longitudinal groin incision; the great saphenous vein was preserved, and the common femoral, proximal deep femoral and proximal superficial femoral arteries were controlled with vessel loops and clamps. Through an arteriotomy at the distal common femoral/proximal superficial femoral artery, organized and fresh red-and-white thrombus was extracted under direct vision and by Fogarty balloon catheter passed proximally (common iliac–external iliac–common femoral) and distally (superficial femoral–common femoral) until vigorous inflow and back-bleeding were obtained (Figure 2). Prior to closure, systemic anticoagulation was maintained with intravenous heparin (100 U/kg bolus administered before cross-clamping, titrated to an activated clotting time >250 s), and the lumen was flushed with heparinized saline. Primary closure without patch angioplasty was selected because the native common femoral artery measured ≥8 mm in diameter. The arteriotomy was then closed primarily with five interrupted 6-0 monofilament polypropylene sutures placed outside-to-in on the proximal lip and inside-to-out on the distal lip to evert the intimal edges and achieve a blood-tight anastomosis. Completion angiography was performed before wound closure to confirm luminal patency and exclude distal embolization.

**Figure 2:**
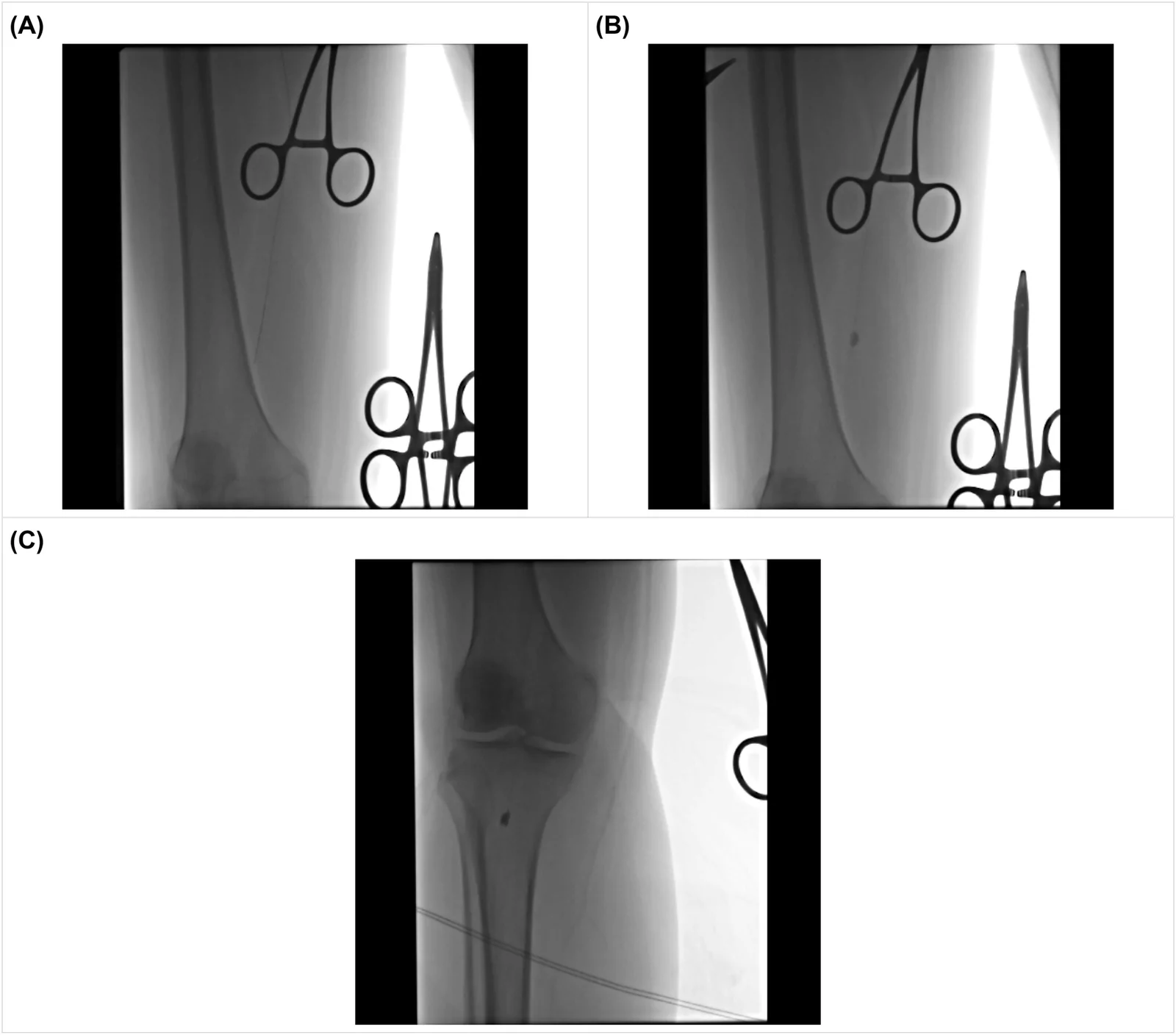
Open surgical thromboembolectomy. **(A,B)** Open right common femoral artery exposure and Fogarty balloon thromboembolectomy (common iliac–external iliac–common femoral proximally; superficial femoral–common femoral distally). **(C)** Intra-operative fluoroscopic view at the knee.

Completion angiography through the MPA catheter confirmed restored flow distal to the right common iliac artery but revealed a flow-limiting dissection of the external iliac and proximal–mid superficial femoral artery. After multidisciplinary discussion and informed consent for stenting, contralateral (left common femoral) retrograde access was obtained (6-F then 7-F crossover sheath) and an ‘up-and-over’ approach used to selectively catheterize the right superficial femoral artery, with true-lumen position confirmed. Two overlapping self-expanding stents (5.5 × 150 mm) were deployed from the distal–mid superficial femoral artery to the distal common femoral artery, and a balloon-expandable covered stent (8.0 × 58 mm) was deployed in the right external iliac artery at 12 atm (Figures 3A–C). Final completion DSA demonstrated widely patent inflow and outflow with a regular luminal contour, no residual flow-limiting dissection or filling defect, abundant collateral filling, and brisk flow reaching the foot (Figure 3D). No on-table thrombolysis was used. The left common femoral puncture was closed with a suture-mediated closure device and the left brachial puncture by manual compression; the groin wound was closed in layers.

**Figure 3:**
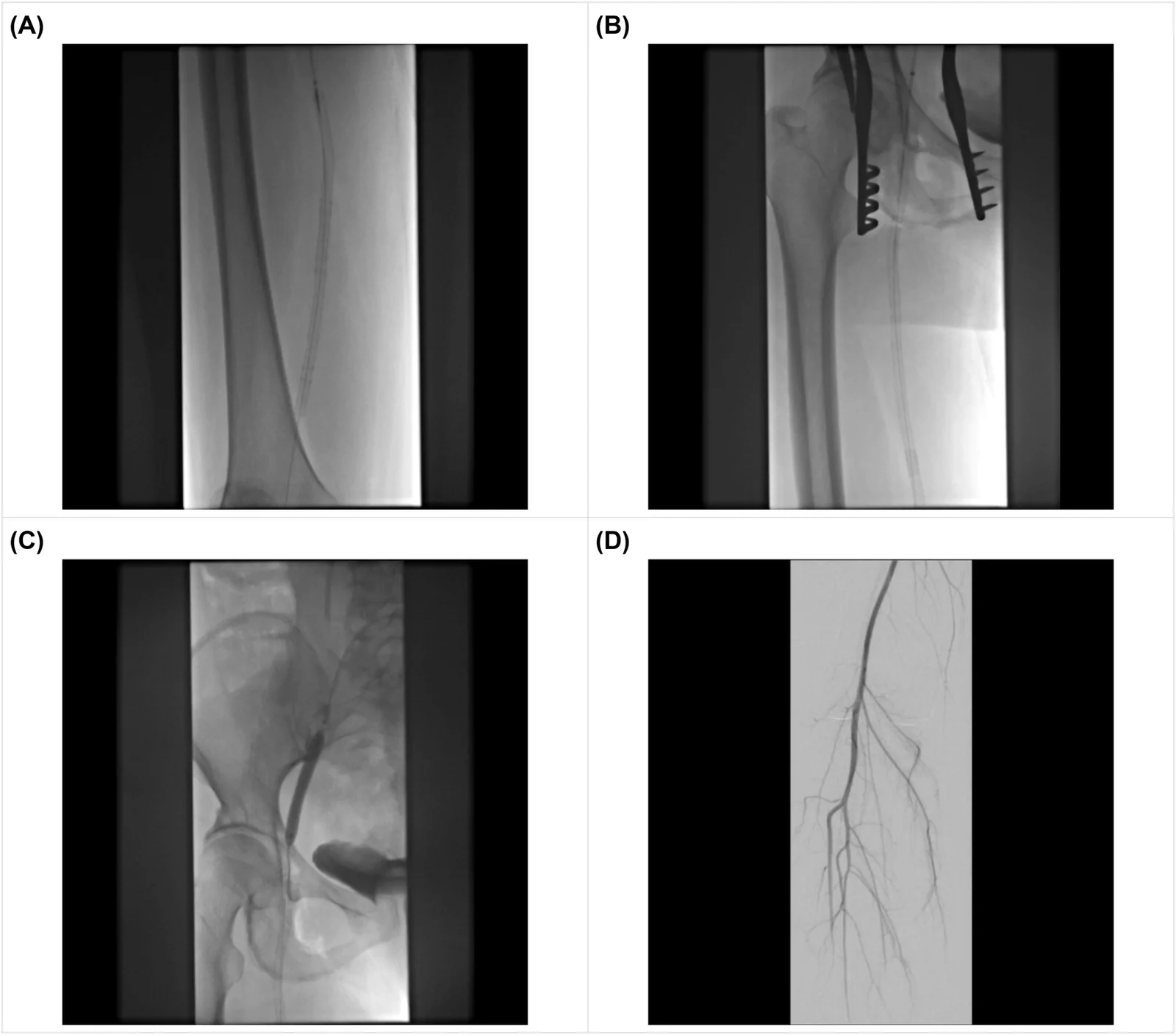
Adjunctive crossover endovascular revascularization and completion angiography. **(A,B)** Crossover access into the right superficial femoral artery after completion angiography disclosed a flow-limiting dissection; **(C)** external-iliac balloon-expandable covered stent deployment (with two overlapping self-expanding superficial-femoral/common-femoral stents); **(D)** completion DSA showing restored tibial/peroneal runoff to the foot.

The retrieved material was submitted in its entirety for histopathological examination, an important step in patients with active malignancy in whom tumor embolism must be distinguished from bland thrombus (8). Grossly, the specimen consisted of mixed red-and-white thrombus with interspersed pale laminated zones, consistent with lines of Zahn (alternating platelet/fibrin and erythrocyte layers). On hematoxylin-eosin (H&E) staining at x100 magnification (Figure 4), two complementary microscopic fields were documented. The first showed a fibrin-rich area with abundant infiltrating neutrophils (small, deeply basophilic, multilobed nuclei) admixed with eosinophilic fibrin strands and a few entrapped erythrocytes, consistent with an acute inflammatory thrombotic component. The second showed a large confluent lake of erythrocytes (intensely eosinophilic) interspersed with fibrin and scattered neutrophils, consistent with the red-cell-rich component of mixed thrombus. Together, the two panels illustrate mixed fibrin-rich and erythrocyte-rich thrombus with prominent acute neutrophilic infiltration and no epithelial cell clusters, glandular architecture, papillary structures, atypical large cells with hyperchromatic or pleomorphic nuclei, or mitotic figures-features of bland (non-neoplastic) thrombus rather than tumor embolism (Figure 4).

**Figure 4:**
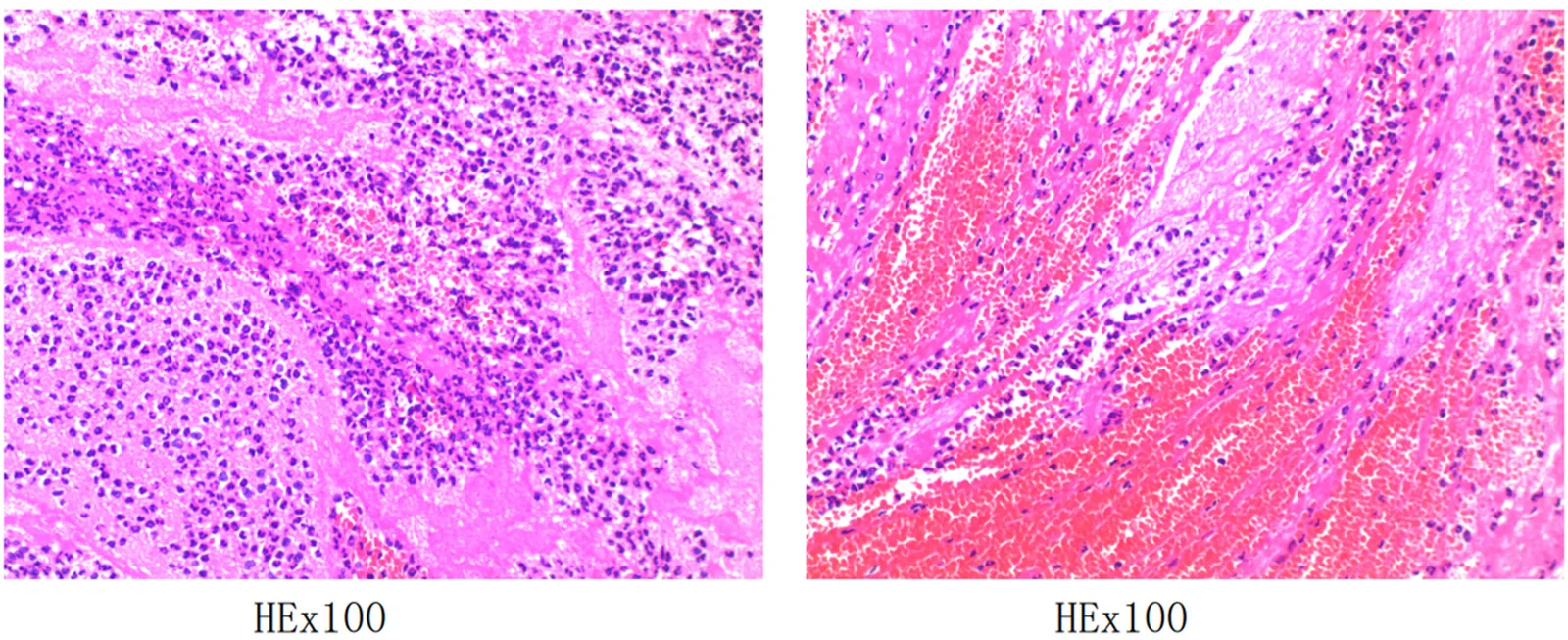
Histopathological characterization of the retrieved thrombus (H&E staining, ×100). (Left panel) A representative field showing a fibrin-rich area with abundant infiltrating neutrophils (small, deeply basophilic, multilobed nuclei) admixed with eosinophilic fibrin strands and a few entrapped erythrocytes, consistent with an acute inflammatory thrombotic component. (Right panel) A complementary field showing a large confluent lake of erythrocytes (intensely eosinophilic) interspersed with fibrin and scattered neutrophils, consistent with the red-cell-rich (so-called “red thrombus”) component of mixed thrombus. Together, the two panels illustrate mixed fibrin-rich and erythrocyte-rich thrombus with prominent acute neutrophilic infiltration and no epithelial cell clusters, glandular architecture, papillary structures, or atypical cells with hyperchromatic or pleomorphic nuclei—features of bland (non-neoplastic) thrombus rather than tumor embolism. Magnifications are nominal.

### Postoperative course and antithrombotic management

Immediately after revascularization the limb was warm and well perfused: the skin colour and temperature of the right lower limb improved markedly, the superficial veins filled, and palpable pulsation returned in the right femoral, popliteal, dorsalis pedis and posterior tibial arteries. The patient's vital signs were stable and she reported no significant discomfort. Therapeutic UFH was continued and then transitioned to a therapeutic, weight-adjusted low-molecular-weight heparin (LMWH); the long-term plan and the choice between continued LMWH and a direct oral anticoagulant (DOAC) were individualized after multidisciplinary discussion among gynecologic oncology, hematology and vascular surgery (see Discussion). Because the patient also received a self-expanding/covered stent construct, the role and duration of adjunctive antiplatelet therapy were weighed against the cumulative bleeding risk. Fuzuloparib was temporarily held peri-procedurally and subsequently resumed after balancing oncologic benefit against thrombotic and bleeding risk, with surveillance of the platelet count given the potential for PARP-inhibitor–associated thrombocytopenia (11, 13).

### Follow-up

At 3 months the patient was with a viable limb, palpable dorsalis pedis and posterior tibial pulses, and a recovered ABI of 0.92 on the affected side (Table 1). The surgical wound had healed without infection, the stents remained patent on duplex, and there had been no recurrent ischemic event, major bleeding or amputation. D-dimer had normalized and CA125 had fallen in line with ongoing oncologic control. Anticoagulation was continued, with a plan for indefinite therapy while the cancer remained active and fuzuloparib was ongoing, subject to periodic reassessment of bleeding risk and renal function (16, 18).

Written informed consent for publication of this report and the accompanying images was obtained from the patient.

## Discussion

This case illustrates ALI occurring at the convergence of several established prothrombotic drivers—most importantly active ovarian malignancy and chronic atherosclerotic disease—superimposed on which PARP-inhibitor exposure may have acted as an additional, speculative contributor, and highlights several clinically important themes: the recognition and mechanism of cancer-associated arterial thrombosis (CAAT), the importance of rigorously excluding a cardioembolic source and tumor embolism before attributing the event, the choice of a hybrid revascularization strategy when underlying stenotic disease is present, and the individualization of long-term anticoagulation despite the absence of dedicated trials in arterial cancer-associated thrombosis.

### Acute limb ischemia in the patient with cancer

ALI in patients with malignancy is uncommon but carries a worse prognosis than in the general ALI population. A systematic review and meta-analysis of outcomes after ALI in cancer (seven studies, 2,899 patients) found that genitourinary, lung, gastrointestinal and skin/soft-tissue cancers accounted for the majority of events and that one-year mortality after ALI exceeded 50% (4). Importantly, the hypercoagulable state of malignancy promotes thrombosis of the distal microvasculature, which can compromise runoff and increase the risk of minor amputation even after technically successful proximal thromboembolectomy (1). Recent reviews of arterial thrombosis in patients with cancer have emphasized that the underlying mechanisms and optimal antithrombotic strategies for cancer-associated arterial events remain substantially less well defined than for cancer-associated VTE, and that data specific to the acute peripheral arterial circulation are particularly limited (30–32). These observations reinforce the need for prompt revascularization, meticulous attention to outflow, and systemic anticoagulation in this population, while recognizing that much of the available evidence is extrapolated from venous or mixed arterial–venous cohorts.

### Mechanisms of cancer-associated arterial thrombosis

Cancer-associated thrombosis is multifactorial (23, 28). Malignant cells express procoagulant molecules—most notably tissue factor, which triggers the extrinsic coagulation cascade, together with podoplanin, plasminogen activator inhibitor-1 and protein disulfide isomerase—and shed tissue-factor–bearing extracellular vesicles into the circulation (6, 7, 27). Mucin–selectin interactions in mucinous adenocarcinomas and the release of neutrophil extracellular traps provide additional scaffolds for thrombus formation (6, 7). In the arterial bed specifically, these mechanisms—together with cancer-related inflammation, platelet activation and, occasionally, non-bacterial thrombotic endocarditis with distal embolization—can produce stroke, visceral infarction and limb ischemia (8). The clinical corollary is that arterial occlusion in a patient with active cancer should not be assumed to be purely atheroembolic; cancer-associated *in-situ* thrombosis must be considered, and histologic examination of the retrieved material is valuable to exclude tumor embolism (8).

### PARP inhibitors and thrombotic risk

PARP inhibitors, including fuzuloparib, exploit synthetic lethality to kill homologous-recombination–deficient tumor cells and have markedly improved progression-free survival in ovarian cancer; in the phase 3 FZOCUS-1 trial, fuzuloparib monotherapy (150 mg twice daily) prolonged median progression-free survival versus placebo (29.9 vs 11.1 months; hazard ratio 0.58), whereas the addition of apatinib conferred no further benefit (13, 14). Their relationship with thrombosis has been increasingly scrutinized. A 2025 systematic review and meta-analysis of 38 randomized trials (15,008 patients) reported only a borderline overall venous thromboembolic signal with PARP inhibitors (any-grade VTE 2.4% vs. 1.6%; odds ratio 1.37, 95% CI 1.00–1.88, *P* = 0.050) that was driven mainly by prostate and pancreatic cancer; the ovarian subgroup (11 trials, 4,348 patients) showed no statistically significant increase (11). Real-world data from the Spanish (SEOM) thrombosis-and-cancer group (329 patients with ovarian cancer) identified BRCA2 mutation as more prevalent among those who developed venous or arterial thromboembolism (56.3% vs. 19.2%), while concomitant bevacizumab was associated with a lower thrombotic risk (odds ratio 0.262, 95% CI 0.095–0.724) (12). Case reports have nonetheless documented pulmonary embolism during PARP-inhibitor maintenance (15). The biological basis for any class effect remains incompletely defined and may involve endothelial PARP-dependent repair, platelet effects and—paradoxically—therapy-related cytopenias. In any individual patient the underlying malignancy is itself a powerful confounder, and causality of an arterial event cannot be inferred from a single case; the contribution of fuzuloparib in this patient should therefore be regarded as speculative and at most additive to a haemostatic milieu dominated by active ovarian cancer and chronic atherosclerosis.

### Diagnostic and biomarker considerations

Several biomarkers framed this case. The ABI provides an objective, reproducible measure of perfusion and recovery, progressing from unobtainable at presentation toward a near-normal value at follow-up (1, 24). D-dimer, although nonspecific, was markedly elevated at presentation and normalized with revascularization and anticoagulation; it is a useful trend marker rather than a diagnostic test in this setting. CA125 serves a dual role as a marker of ovarian-cancer activity and, indirectly, of prothrombotic burden, and its trend tracks oncologic control. The Rutherford classification remained the central tool for triaging the limb and selecting therapy (1, 2). Two diagnostic steps are central to a credible attribution of the event to *in-situ* cancer-associated arterial thrombosis rather than to cardiac embolism or tumor embolism, and were explicitly performed here: first, systematic exclusion of a cardioembolic source—through 12-lead ECG, continuous perioperative telemetry, transthoracic echocardiography (no intracardiac thrombus, no valvular vegetation, no mass, preserved biventricular function) and carotid duplex (no critical stenosis); second, histopathological characterization of the retrieved thrombus, which showed bland fibrin–platelet–red-cell thrombus with lines of Zahn and negative AE1/AE3 immunostaining, thereby excluding tumor embolism (30, 32). These two steps substantially strengthen the inference of cancer-associated *in-situ* arterial thrombosis on a background of chronic atherosclerosis.

### Revascularization strategy: why a hybrid approach

For Rutherford II ALI, revascularization must be both rapid and complete. The principal options are open surgical thromboembolectomy, catheter-directed thrombolysis, percutaneous mechanical/aspiration thrombectomy, and hybrid combinations (1). Open Fogarty thromboembolectomy offers immediate clot removal and is well suited to an immediately threatened limb, whereas endovascular techniques are advantageous for treating residual thrombus and underlying stenotic lesions and for reaching the distal runoff. In this patient the two were combined: open femoral cutdown achieved prompt proximal and distal clot extraction, while intra-operative completion angiography disclosed a residual flow-limiting dissection and underlying disease that were addressed by contralateral crossover access with stenting in the same setting. The ESVS guidelines acknowledge that combined open and endovascular (hybrid) treatment is useful when residual stenosis or thrombus is present after thromboembolectomy, and they highlight completion angiography as a key adjunct (1); routine intraoperative imaging with selective endovascular adjuncts has been associated with high technical success and limb salvage in contemporary native-vessel ALI series (29). A hybrid approach is particularly attractive when cancer-associated thrombosis is superimposed on chronic atherosclerosis, where optimization of inflow/outflow and avoidance of residual thrombus or dissection are especially important (1, 4).

### Long-term anticoagulation strategy

The most challenging aspect of management is the long-term antithrombotic plan, which must reconcile a very high thrombotic risk with competing bleeding and drug-interaction concerns. Contemporary guidelines for cancer-associated thrombosis (ITAC 2022, ASCO 2023, ASH 2021, NCCN 2021) recommend LMWH or a DOAC for at least three to six months and—critically—suggest extending anticoagulation, potentially indefinitely, while the cancer remains active or the patient continues systemic therapy, with periodic reassessment (16–18, 26). The pivotal randomized trials underpinning DOAC use in cancer-associated VTE—Hokusai-VTE Cancer (edoxaban), SELECT-D (rivaroxaban) and Caravaggio (apixaban)—demonstrated non-inferiority to dalteparin for recurrent VTE, although edoxaban and rivaroxaban were associated with more gastrointestinal/genitourinary bleeding, whereas apixaban showed no significant excess of major bleeding (20–22); the earlier CLOT trial established LMWH (dalteparin) superiority over warfarin (19). Two caveats are pertinent here. First, DOACs (factor Xa inhibitors) are substrates of CYP3A4 and P-glycoprotein and may interact with anticancer and psychotropic agents (this patient also received venlafaxine and other CNS-active drugs); potential pharmacokinetic interactions and renal function should be reviewed before and during DOAC use (16, 25). Second, DOACs are associated with a higher risk of gastrointestinal and genitourinary bleeding, directly relevant to a patient with pelvic ovarian disease; in such patients LMWH is often preferred, at least initially (16, 18). PARP-inhibitor–associated thrombocytopenia adds a further dimension, since the anticoagulant dose may need modification according to the platelet count, with multidisciplinary input (11, 13, 18).

A systematic cross-check of high-quality guidelines directing cancer-associated arterial versus venous thrombosis is informative, and reveals an important evidence asymmetry. The four principal contemporary practice guidelines for cancer-associated thrombosis—ITAC 2022 (16), ASCO 2023 (17), ASH 2021 (18) and NCCN 2021 (26)—were developed primarily for, and explicitly target, venous thromboembolism (lower-extremity deep-vein thrombosis, pulmonary embolism, splanchnic-vein thrombosis and catheter-associated thrombosis); none provides a dedicated, evidence-graded recommendation set for cancer-associated arterial thrombosis of the lower limb, and management decisions for arterial events must therefore be extrapolated from VTE-derived recommendations or from non-cancer peripheral arterial disease guidelines (which in turn do not address the unique bleeding and drug-interaction profile of malignancy). The recently published focused reviews on cancer-associated arterial events (30–32) are explicitly framed as narrative syntheses and expert-opinion–based recommendations, not as guidelines, and they consistently emphasize that the arterial evidence base consists predominantly of small retrospective series, registry analyses and subgroup extrapolations, with very few randomized data. The consequence is a double extrapolation: not only from VTE to arterial events, but also from non-cancer peripheral arterial disease into cancer-associated arterial disease. Two specific clinical implications follow. First, agent selection (LMWH vs. DOAC vs. VKA) is currently justified on VTE evidence, with the bleeding-risk signal (more gastrointestinal/genitourinary bleeding with rivaroxaban/edoxaban than with dalteparin; no clear excess major bleeding with apixaban) extrapolated to a population with possible pelvic/genitourinary sources. Second, no guideline-anchored duration of therapy exists for cancer-associated ALI; the suggestion of “indefinite” anticoagulation while the cancer remains active is, again, an extrapolation from VTE guidance. Until tumor-/arterial-bed–specific trials are available, the present case was managed pragmatically by extending LMWH through the early post-revascularization phase, with a plan to consider transition to apixaban (the DOAC with the most favorable bleeding profile in cancer-VTE trials and the least pharmacokinetic interaction with the patient's outpatient medications) at 3 months, subject to bleeding risk, renal function, platelet count and continued oncologic activity.

It must be acknowledged that the high-quality evidence underpinning these recommendations derives largely from venous thromboembolism; data specific to cancer-associated arterial events such as ALI are sparse, and management is necessarily extrapolated and individualized. Some authorities favour therapeutic LMWH for cancer-associated arterial thrombosis, and the role of adjunctive antiplatelet therapy—particularly when an atherosclerotic component or endovascular stenting is involved, as here—must be balanced against the cumulative bleeding risk (8, 25). In the present case the patient was maintained on therapeutic anticoagulation with a plan for indefinite treatment while the malignancy and PARP-inhibitor therapy continued, and with structured surveillance of perfusion, platelet count, renal function and bleeding. This pragmatic, multidisciplinary approach reflects the current balance of evidence and the absence of dedicated trials in this specific population.

It must be acknowledged that the high-quality evidence underpinning these recommendations derives largely from venous thromboembolism; data specific to cancer-associated arterial events such as ALI are sparse, and management is necessarily extrapolated and individualized. Some authorities favour therapeutic LMWH for cancer-associated arterial thrombosis, and the role of adjunctive antiplatelet therapy—particularly when an atherosclerotic component or endovascular stenting is involved, as here—must be balanced against the cumulative bleeding risk (8, 25). In the present case the patient was maintained on therapeutic anticoagulation with a plan for indefinite treatment while the malignancy and PARP-inhibitor therapy continued, and with structured surveillance of perfusion, platelet count, renal function and bleeding. This pragmatic, multidisciplinary approach reflects the current balance of evidence and the absence of dedicated trials in this specific population. Detailed supporting evidence on cancer-associated arterial thrombosis, PARP-inhibitor thrombotic risk and anticoagulation guidance is summarized in Table 2.

### Limitations

This is a single case, and causal attribution of the arterial event to fuzuloparib as distinct from the underlying malignancy and pre-existing atherosclerosis is not possible. Several limitations of the present report merit explicit acknowledgment. First, the follow-up window is limited to 3 months, which is shorter than the timeframes typically required to assess the durability of endovascular reconstructions and the long-term bleeding–thrombosis balance of indefinite anticoagulation in patients with active malignancy: in contemporary endovascular series, clinically significant in-stent restenosis, late stent thrombosis and re-occlusion accumulate predominantly between 6 and 24 months, and major bleeding on extended anticoagulation most often occurs after the first 3–6 months. The current 3-month data therefore demonstrate early anatomic and functional success but cannot establish long-term stent patency, nor the cumulative incidence of major adverse cardiovascular events (MACE/MALE), clinically significant bleeding (ISTH major or CRNM bleeding), recurrent thromboembolism, or amputation-free survival. Second, the patient's cancer trajectory (progression of ovarian cancer, second-line chemotherapy, hospice transition) may itself alter the bleeding–thrombosis risk profile and the optimal antithrombotic intensity, and was not captured within the present window. Third, no formal Khorana or CATScore assessment was performed at baseline or during follow-up to provide a quantified risk estimate. Fourth, platelet-function testing, anti-Xa monitoring for LMWH, and serial D-dimer trends were not available across the full 3-month period. The optimal agent and duration of anticoagulation, the role and duration of adjunctive antiplatelet therapy in the presence of both atherosclerotic disease and a covered/self-expanding stent construct, and the comparative bleeding–thrombosis balance of LMWH versus DOAC in cancer-associated arterial thrombosis can therefore only be resolved by larger, ideally prospective and tumor-/arterial-bed–specific, studies with follow-up of at least 12–24 months.

## Conclusions

ALI in a patient with active ovarian cancer and chronic atherosclerotic disease—on a background that also included PARP-inhibitor exposure—reflects a profoundly prothrombotic state and demands prompt, complete revascularization. Any independent contribution of the PARP inhibitor to the arterial event remains speculative and cannot be inferred from a single case; active malignancy and atherosclerosis are themselves sufficient to explain the *in-situ* thrombus. A hybrid strategy combining open femoral thromboembolectomy with completion angiography and contralateral crossover endovascular stenting achieved durable limb salvage in a single setting. Beyond the operation, the central management question is long-term anticoagulation: clinicians should treat the patient as high-risk for recurrence, exclude a cardioembolic source and tumor embolism (the latter histologically), and individualize extended anticoagulation—largely extrapolated from cancer-associated VTE evidence and guidelines, with explicit acknowledgment of the arterial-evidence gap, and with attention to drug–drug interactions, thrombocytopenia and pelvic/genitourinary bleeding risk—within a multidisciplinary framework. Heightened awareness of cancer-associated arterial thrombosis is warranted in this population; further prospective, ideally tumor- and arterial-bed–specific, studies are needed to clarify the optimal agent, duration and combination of antithrombotic therapy.

## Data Availability

The original contributions presented in the study are included in the article/Supplementary Material, further inquiries can be directed to the corresponding author.
